# UV-Denaturation Assay to Assess Protein Photostability and Ligand-Binding Interactions Using the High Photon Flux of Diamond B23 Beamline for SRCD

**DOI:** 10.3390/molecules23081906

**Published:** 2018-07-31

**Authors:** Rohanah Hussain, Edoardo Longo, Giuliano Siligardi

**Affiliations:** B23 Beamline, Diamond Light Source, Harwell Science Innovation Campus, Chilton, Didcot OX11 0DE, UK; edoardo.longo@diamond.ac.uk

**Keywords:** circular dichroism, ligand binding, high photon flux, protein stability, synchrotron radiation, SRCD, vacuum UV

## Abstract

Light irradiation with high photon flux in the vacuum and far-UV region is known to denature the conformation of biopolymers. Measures are in place at Diamond Light Source B23 beamline for Synchrotron Radiation Circular Dichroism (SRCD) to control and make this effect negligible. However, UV denaturation of proteins can also be exploited as a novel method for assessing biopolymer photostability as well as ligand-binding interactions. Usually, host–ligand binding interactions can be assessed monitoring CD changes of the host biopolymer upon ligand addition. The novel method of identifying ligand binding monitoring the change of relative rate of UV denaturation using SRCD is especially important when there are very little or insignificant secondary structure changes of the host protein upon ligand binding. The temperature study, another method used to determine molecular interactions, can often be inconclusive when the thermal effect associated with the displacement of the bound solvent molecules by the ligand is also small, making the determination of the binding interaction inconclusive. Herein we present a review on the UV-denaturation assay as a novel method to determine the relative photostability of protein formulations as well as the screening of ligand-binding interactions using the high photon flux Diamond B23 beamline for SRCD.

## 1. Introduction

Circular Dichroism (CD) is a spectroscopic technique to obtain low-resolution structural information about a wide variety of chiral materials in solution such as small molecules (drugs), proteins, DNA, and polymers. For biopolymers, the knowledge of their active conformation and their conformational behaviour as a function of environment (solvent polarity, pH, temperature, chemical agents, and detergents) are essential to understand the mode of action at the molecular level and identify quickly new potential targets for novel therapeutic drugs [1,2,3,4,5,6,7,8,9,10,11,12,13].

In the far-UV region, benchtop CD instruments using arc Xe lamps possess low photon flux at shorter wavelength than 200 nm, requiring repeated consecutive scans and/or increased integration time to achieve good signal-to-noise spectra. The use of synchrotron radiation light overcame this limitation, extending the utility of the technique down 130 nm in the vacuum-UV region [14,15]. The development of Diamond B23 beamline for synchrotron radiation circular dichroism (SRCD) with a unique, highly collimated microbeam [6] enabled the use of small aperture cuvette cells and capillaries (flat or round) to study precious samples with limited availability otherwise unattainable with benchtop CD instruments [8].

Diamond Light Source B23 beamline for SRCD has been designed to generate high photon flux (at 200 nm 3.2 × 10^12^ photons s^−1^ (0.1% bandwidth)^−1^) [6]. Although high UV photon flux leads to protein denaturation in the far-UV region [7,8], the implementation of a set of simple yet effective measures enables the control of the denaturation/degradation of biomolecules which can be used as a novel assay to assess biopolymer photostability and ligand-binding interactions.

We have previously reported the protein photo denaturation using the high far-UV photon flux of B23 beamline [7]. Like thermal denaturation, UV denaturation varies from protein to protein showing different amounts of conformational changes that correlate with the degree of protein stability. With synchrotron beamlines for SRCD, the protein photo denaturation induced in far-UV region is radiation power and dose-dependent [16]. This effect can also be induced using Chirascan CD spectropolarimeter (Applied Photophysics, Leatherhead, UK) but with bandwith ≥4 nm. However, as the photon flux generated by the Xe arc lamp of the Chirascan decreases with the age of the lamp, the effect is greater with new lamps and greatly reduced for old lamps. Also, the damage of the monochromator MgF_2_-coated aluminium mirrors is increased when operating the instrument with bandwidths greater than 1 to 2 nm [16]. This limitation does not exist for B23 as the beamline operates in constant topping mode between 290 and 300 mA [6] and the mirrors are made of uncoated silicon to withstand any solarisation effects.

OH radicals from water radiolysis formed by UV irradiation of aqueous solutions have been proposed as the cause of the protein unfolding, termed UV denaturation [17]. Another mechanism has been suggested where the thermal effect of the water molecules bound to the proteins when irradiated with far-UV light was the cause of protein UV denaturation [18,19]. This latter mechanism was based on the apparent similarity of the protein conformational changes induced by both UV irradiation and heating. The fact that under the same conditions—number of scans and beamline configuration in the far-UV region—the protein UV denaturation does not occur in the near-UV region (250–330 nm) (data not shown) even though the thermal denaturation can still be observed in this wavelength range, is a further indication that the origin of the protein UV denaturation is not due to thermal effects. However, as the SRCD changes are UV radiation and dose-dependent, namely irradiation time, the protein denaturation in the near-UV region cannot be ruled out for very long light exposures.

The good news is that SRCD protein UV denaturation/degradation at B23 can be controlled.

## 2. Discussion

### 2.1. Exploitation and Control of the High UV Photon Flux of B23

**Figure 1:**
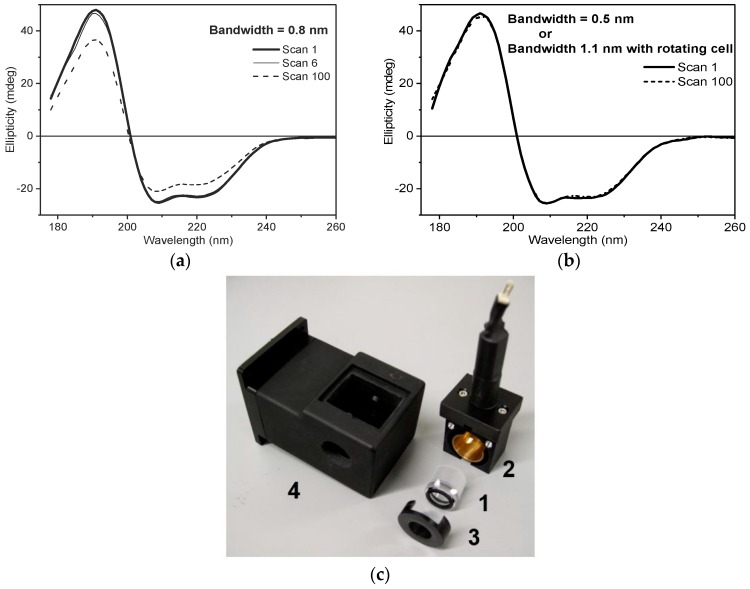
100 Repeated CD scans of HSAff in H_2_O measured with B23 end-station without plano-convex lens, integration time = 1 s, 0.02 cm cylindrical cell, and with different monochromator slit widths. (**a**) With 0.280 mm slit (0.8 nm bandwidth (bw)), only the first six spectra were within 3% of the overall intensity change whilst with (**b**) 0.200 mm slit (bw = 0.5 nm), no significant denaturation was observed to 100 repeated scans. Similar result was observed when a rotating cell holder (photo below) with 1.1 nm bw was used. (**c**) Rotating cell holder: 1, cylindrical cell; 2, cell holder with rotor; 3, cover; and 4, Peltier temperature controlled holder.

For B23, the control of the UV denaturation can be achieved by adopting a combination of different measures such as:

1. Increased irradiated area of the sample by removing the lens located in front of the polariser Rochon prism [6]. This action decreases the photon flux density (brilliance), increasing the protein stability against UV denaturation.
2. Decreased photon flux by reducing the slit width of the double grating subtractive monochromator from 0.500 mm to 0.200 mm, which correspond to a bandwidth of 1.1 and 0.5 nm, respectively.
3. Combination of the above two measures as illustrated in Figure 1 where the least photostable human serum albumin essentially fatty-acid-free and globulin-free (HSAff) can withstand the far-UV irradiation of 100 consecutive scans.
4. Using the rotating cell holder for cylindrical cells (Hellma 121.00-QS type) (Figure 1). In this manner, the monochromator slit width can be kept as wide as possible yet allowing a high number of consecutive spectra (up to 100 scans) to be scanned without inducing detectable CD spectral changes.

In general, it is not necessary to scan up to 100 SRCD spectra. Often, 20–30 repeated consecutive scans are sufficient to assess the degree of protein photostability and what measure or measures are more appropriate to eliminate it. For studies where protein conformational changes may occur in the far-UV region as a function of temperature and/or ligand-binding interactions, it is essential, however, that the protein UV denaturation is eliminated. In these cases, the SRCD changes will arise only from the chosen type of perturbing agent such as temperature, pH, and ligand titration, and not from the contribution of UV denaturation.

### 2.2. Temperature Scan

For example, an experiment where the SRCD is measured every 2 °C would require 46 spectra for the 4–94 °C heating ramp and 45 spectra for the reversed 92–4 °C cooling ramp for a total of 91 spectra. If three repeated scans are required for each temperature increment to improve the signal-to-noise ratio, a compromise for a 5–85 °C range at 5 °C increments rather than 2 °C has to be made, resulting in 99 scans. For these studies, it is recommended to operate in scanning mode rather than at fixed wavelength. With the scanning mode, the plot of the CD intensity versus temperature can be extracted slicing the CD spectra at the wavelength of the most significant spectral features, usually for proteins at 190 nm, 210 nm, and/or 220 nm wavelengths. The advantage of measuring the full CD spectrum rather than the change at fixed wavelength is that for each temperature, the secondary structure content can be estimated, revealing at which temperatures the α-helix, β-strand, polyproline of type II (PPII), β-turn, and unordered elements are varying: increasing, decreasing, or remaining stable often at the expense of each other [20,21,22]. We strongly recommend that the study of protein behaviour is richer in information if conducted measuring the full spectrum rather than the fixed wavelength as a function of the perturbing agent. 

### 2.3. Titration

The CD titration is another example that requires the recording of many spectra to determine the formation of molecular interactions. Although the observation in the far-UV region of SRCD spectral changes of the protein secondary structure upon the addition of the ligand is unambiguously indicative of binding interactions, the contrary is not true. As long as a molecular interaction does not change the secondary structure content or induce any distortion of the canonical secondary structures, the lack of detectable CD changes does not necessarily mean that there are no binding interactions. Though this is often the case, binding interactions that affect the quaternary structures can take place without significant changes at the protein secondary structure level. For proteins, the CD in the far-UV region is mainly originated by the coupling of the electric transition moments of the amide bond of π–π*. Unordered structures like loops possess are characterized by a weak and U type of spectrum centered at about 200 nm. Changes of the dihedral angles of these loops will generate very little CD changes. Therefore, in a protein “breathing” mode, the movements of structured domains around loops will be largely invisible in the far-UV but could be seen in the near-UV region that is sensitive to the local environment of the side-chain of aromatic residues and dihedral angle of disulphide bonds [9,10,11].

Very often, the near-UV region offers the best choice of detecting CD changes of the local environment of aromatic amino-acid residues upon ligand addition that can be used successfully as natural probes to determine ligand-binding interactions qualitatively and quantitatively using a nonlinear regression analysis [8,9,10,11]. On the other hand, if the aromatic chromophores of the protein residues or the ligand are far from the binding site (>6 Å) or the ligand is devoid of suitable chromophores, other methods to determine molecular interactions must be used, such as the temperature study discussed above (thermal stability often increases with protein–ligand complex formation) or isothermal calorimetry (ITC). The use of ITC could have limitations due to the large amount of sample required in comparison with CD studies or the fact that the heat involved in the binding interactions is rather small or compensated by the replacements of many bound water molecules.

### 2.4. UV Photodenaturation Application

**Figure 2:**
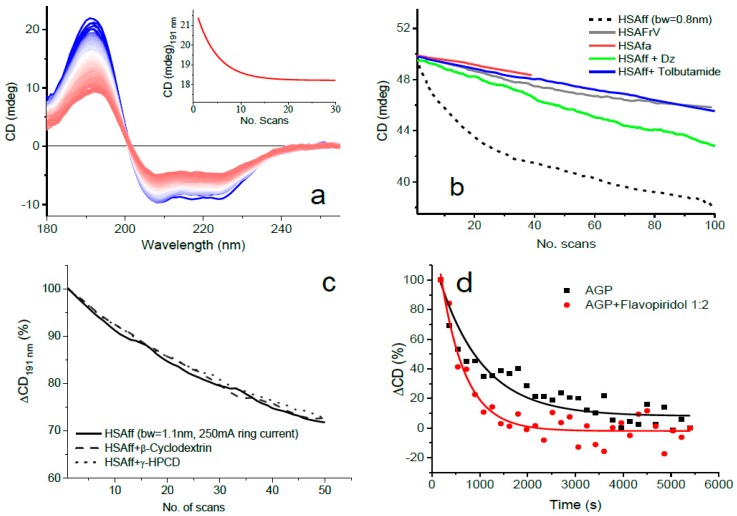
(**a**) Thirty repeated consecutive SRCD spectra of HSAff with inset the rate of protein denaturation at 191 nm. (**b**) Plot of SRCD percentage intensity change relative to the first scan (∆CD) at 191 nm versus the number of scans for HSAff in H_2_O (10 µM) and with ligands such as diazepam (DZ), tolbutamide, and fatty acids (fa). The solutions with fatty acids were respectively labelled HSAfa and HSAfrV based on the different amounts of octanoic acid (redrawn from [7]). (**c**) Negative control with β-cyclodextrin, γ-hydroxypropyl cyclodextrin, and HSAff. (**d**) acid-glycoprotein (AGP) in the presence of antileukaemia agent, flavopiridol.

In these cases, the protein denaturation induced by UV irradiation provides a novel type of assay to determine the biopolymers’ stability and ligand-binding interactions. Figure 2 illustrates the spectral changes of HSAff with and without ligands such as diazepam (1:1 molar ratio), fatty acids (octanoic acid), tolbutamide, β-cyclodextrin, γ-cyclodextrin, and flavopiridol when irradiated through repeated consecutive scans. The repeated spectra were scanned under the same conditions of integration time = 1 s, bandwidth = 0.8 or 1.1 nm, wavelength range = 260–178 nm, synchrotron radiation ring current of 250 mA, HSAff concentration (5 µM or 10 µM in a 0.02 cm cell), and number of scans (100) as for HSAff alone reported in Figure 1. The reduced overall intensity of the SRCD spectra was indicative of a decreased content of α-helical conformation that was greater for albumin alone (Figure 2a) than for albumin with the ligand (Figure 2b).

#### 2.4.1. Ligand Binding

With the UV-denaturation assay, the protein HSAff showed substantially higher photostability with ligands than without that can be used successfully to identify qualitatively binding interactions, particularly for ligand devoid of UV absorption in the far-UV region (Figure 2b). 

The rate of protein UV denaturation is better illustrated in the plot of the SRCD intensity at fixed wavelength (191 nm) versus the number of scans that shows different kinetics (Figure 2b). The initial slope of protein denaturation was much steeper for human serum albumin fatty acid free (HSAff) than the other types of albumin–ligand mixtures (HSAfa and HSAfrV) that correlated with an increased stability due to the binding interactions with Diazepam, Tolbutamide, and octanoic acid. It is interesting to note that a similar stability was observed for two types of albumin, HSAfa and HSAfrV, that contain approximately 4 and 2 equivalents of octanoid acid, respectively. As an example of a negative control using β-cyclodextrin (β-CD) and γ-hydroxypropyl cyclodextrin (γ-HPCD), they were also measured in a mixture with HSAff 1:1, which showed no changes in the rate of UV denaturation at 191 nm (Figure 2c). Flavopiridol is used as potential antileukaemia and showed to bind to acid glycoprotein, which is the next most abundant circulating protein in the blood, altering the photodenaturation profile of AGP (Figure 2d). 

**Figure 3:**
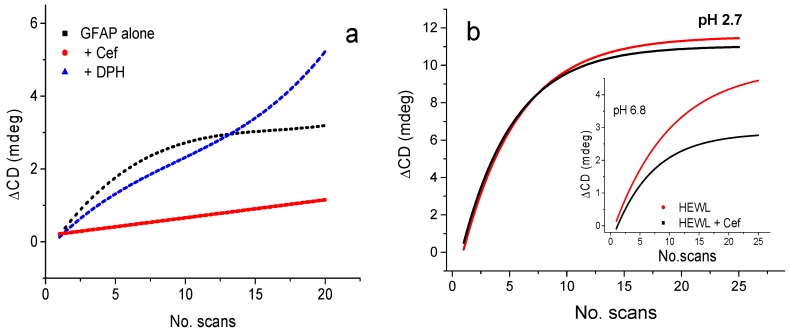
UV photodenaturation plot of change in CD intensity (∆CD) at 191 nm as a function of number of scans. (**a**) Glial fibrillary protein (GFAP) in the presence of 2 stoichiometric ration equivalent of ceftriaxone (Cef) and phenytoin to GFAP and HEWL (redrawn from [27]), (**b**) Hen lysozyme with and without Cef pH at 2.7, and (inset) at pH 6.8 (redrawn from [28]).

It is not trivial to determine the binding of achiral molecules or those with carbonyl chromophores such as sugar, for example, glycosaminoglycans (GAGs), that have little or no absorption or CD signal contributions in the far-UV region [23]. GAGs are present in all amyloid deposits. It is known that the interaction between lysozyme, a prototypic amyloid-forming protein, and GAGs can be altered by the addition of cations [23], which might have implications in the formation of amyloid. We have also recently reported the use of UV-denaturation assay to probe the mechanism of amyloidosis of α-synuclein [24,25,26], glial fibrillary protein (GFAP), and human lysozyme [27,28,29]. The use of β-lactam antibiotic ceftriaxone as a potential drug to ameliorate neurodegenerative diseases was studied using GFAP and lysozyme showing that the presence of ceftriaxone could reduce significantly the rate of UV photodenaturation of the proteins (Figure 3a).

Ceftriaxone has been found to interact with GFAP (Figure 3a), one of the proteins involved in specific neurodegenerative diseases, inhibiting their pathological aggregation [24,25,26,27]. Ceftriaxone may act as a chaperone-like molecule, preventing or reversing protein denaturation and aggregation [28]. The effect of ceftriaxone was found to be pH-dependent. In phosphate buffer, pH 6.8, ceftriaxone appeared to decrease the UV-induced lysozyme denaturation (Figure 3b), whilst in Gly buffer pH 2.8, no significant effect of ceftriaxone was detected (Figure 3b) [28,29].

**Figure 4:**
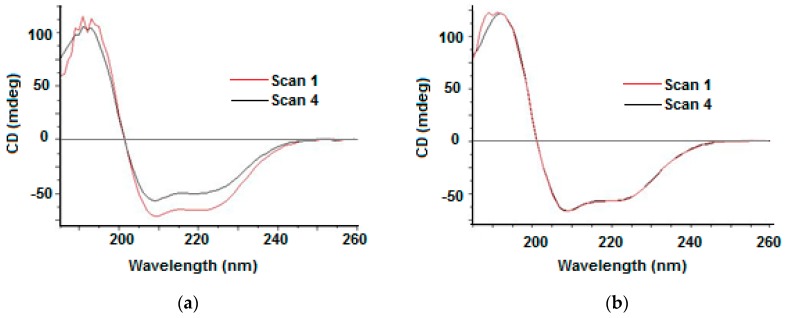

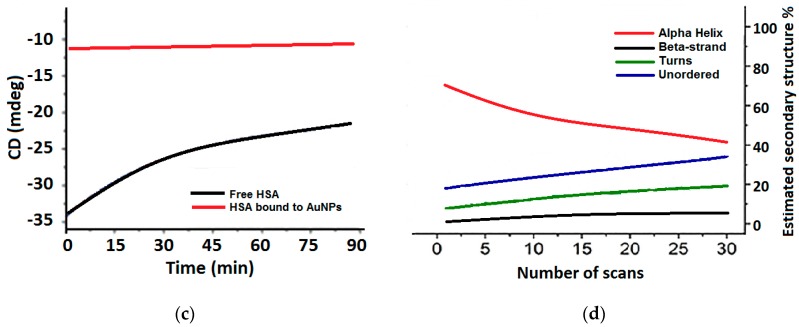
(**a**) Four repeated consecutive SRCD spectra of free HSA: first scan in red and fourth scan in black. (**b**) Four repeated consecutive SRCD spectra of HSA-AuNP system: first scan in red and fourth scan in black. (redrawn from [31]). (**c**) Secondary structure content estimated for the free HSA from the 30 repeated consecutive scans. (**d**) The SRCD photodenaturation assay of HSA (red circles) and HSA bound to AuNP (black circles) was conducted as 30 consecutive repeated scans. As each spectrum was measured in 3 min, the overall exposure of the 195–250 nm wavelength range could be approximated as 90 min. The SRCD intensity at 209 nm is plotted against overall irradiation time (free HSA fitted to a single exponential function, blue line and data for HSA bound to AuNPs fitted with a linear function, orange line). In this plot, the first SRCD values at 209 nm of the first spectrum is the “zero” time irradiation.

Another example of the application of the UV-denaturation assay is that it revealed with few repeated consecutive SRCD scans the photostabilising effect of gold nanoparticles (AuNP) on human serum albumin (HSA) (Figure 4a,b). This can be illustrated in the plotting of SRCD intensity at 209 nm versus irradiation exposure time (Figure 4c) and the loss of α-helical content induced by UV irradiation that was mirrored by the increase of β-strand, β-turn, and unordered conformations (Figure 4d) [30,31,32,33].

#### 2.4.2. Peptide Design

**Figure 5:**
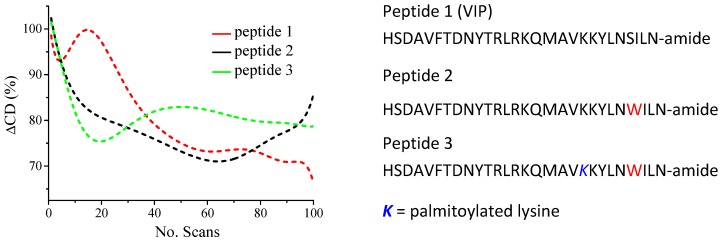
Percentage change in ellipticity at 190 nm versus number of scans for the VIP and two analogue peptides in Tris buffer, 20 mM, pH 7.5, containing 25 mM DPC (redrawn from [33]).

The conformational changes promoted by perturbations of the environment or modifications of the primary sequence can affect both the physical and biological properties of peptides and proteins [9,33]. In the case of vasoactive intestinal peptide (VIP) and VIP-analogues, the acylation of amino groups of the peptide sequence appeared to induce different conformations in aqueous solutions [33]. In membrane-like environments, however, no conformational changes were observed [33]. As VIP is known to bind to G-protein coupled receptor (GPCR), a membrane protein, the understanding of the local binding environment is important as it might give further insight into the structure activity relationship of VIP and guiding the design of more active VIP analogues. Increasing the VIP α-helical conformation in the analogues was crucial as it appeared to enhance the biological activity. While all three peptides lost similar amounts of helical contribution after 100 scans, VIP is more resistant over the first 20 scans compared to the other two peptides before reaching a plateau as shown in Figure 5.

#### 2.4.3. Bioformulation

**Figure 6:**
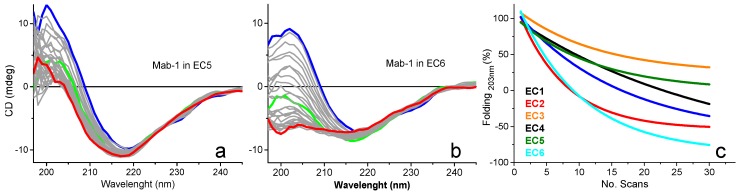
(**a**) 30 far-SRCD spectra of a monoclonal antibody Mab1 in EC5, one of six different formulations (EC1 to EC6) (1st scan (blue); 12th scan (green); 30th scan (red)) (redrawn from [9]). (**b**) 30 far-SRCD spectra of a monoclonal antibody Mab1 in EC6 (redrawn from [9]). (**c**) Rates of UV denaturation of Mab1 in 6 different formulations, EC1 to EC6, showing that Mab1 in EC4 (100% to 70%) was clearly the most stable formulation of the six studied (redrawn from [9]).

An application of the UV-denaturation assay is the screening of formulations that promote protein stability [9]. The efficiency of the screening was increased by using the 6-cell turret. The rate of UV denaturation of a monoclonal antibody Mab1 in six different formulations was determined for each formulation by scanning in the far-UV region (185–260 nm) 30 consecutive repeated spectra that corresponded to an irradiation dose of approximately 90 min (Figure 6). For each formulation, 30 repeated consecutive SRCD spectra revealed the collapse at different rates of the positive CD band at about 200 nm associated with the π–π* transition of the β-strand conformation, which was the direct indication of the loss of secondary structure as the antibody unfolded and was related to the reduction of protein stability. The complete experiment was carried out overnight over 9 h as a single multiscript experiment controlling the operation of the B23 beamline. The six rates of UV-denaturation assay revealed that Mab1 in buffer formulation EC4 was the most stable (100% to 70%, Figure 6c) and the least stable in EC6 formulation (Figure 6b). In terms of relative stability, the six formulations can be therefore ranked qualitatively as follows: EC4 > EC5 > EC1 = EC2 >> EC6 (Figure 6c) [9].

#### 2.4.4. Food Chemistry

**Figure 7:**
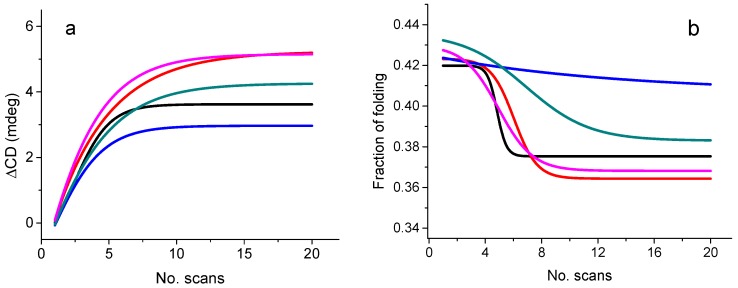
(**a**) Plot of the change in SRCD signal (ΔCD) at 195 nm for VVTL1 (black, IS = 0.859); VVTL1 + C6 (red, IS = 0.957); VVTL1 + C8 (blue, IS = 0.748); VVTL1 + C10 (cyan, IS = 0.799); and VVTL1 + C12 (pink, IS = 1.05) versus number of scans. For each sample, ‘‘IS” is the value of the initial slope of the curve. (**b**) Plot of β-sheet content for VVTL1 (black); VVTL1 + C6 (red); VVTL1 + C8 (blue); VVTL1 + C10 (cyan); and VVTL1 + C12 (pink) determined with CONTINLL [18] of CDApps [39] from SRCD data versus number of scans. For all measurements, protein/ester molar ratio was 1:4 (redrawn from [37]).

Another relevant application of the use of the 6-cell turret was the ligand screening of ethyl esters interacting with wine proteins. During wine fermentation, ethyl esters, which largely contribute to the wine aroma, have been suggested to interact with wine proteins [34]. In the production of wine, bentonite is used to stabilize the wine. However, an undesired effect of the bentonite treatments is the removal of wine proteins that bound to the aroma alkyl esters, altering the wine aroma negatively [35,36]. The UV-denaturation assay carried out at B23 beamline [37] confirmed that the main wine protein, the thaumatin-like protein VVTL1, was able to bind to ethyl esters of different chain lengths: C6, C8, C10, and C12 (Figure 7). The rates of UV denaturation of 20 consecutive repeated SRCD scans for the VVTL1 protein in the presence of alkyl esters were significantly different from that of VVTL1 alone and these results were unambiguously indicative of the formation of protein–ethyl ester complexes. Ethyl octanoate (C8) appeared to increase the VVTL1 photostability more than the other C6, C10, and C12 esters with the following order: C8 > C10 > C12 ≈ C6 (Figure 7a).

The content of β-strand and unordered conformations estimated from each of the 20 repeated consecutive SRCD scans was different for the complexes of VVTL1 with ethyl octanoate (VVTL1-C8) and VVTL1 with ethyl decanoate (VVTL1-C10) than that of VVTL1 alone (Figure 7b). Among the protein–ethyl ester complexes, the one with octanoate (C8) showed very slight UV-denaturation rate, whilst that with C6 showed the highest rate, suggesting that this ester may act as a negative effector for the protein stability. The results of the UV-denaturation assay indicated that the treatment with bentonite should be carried out before the aroma compounds (ethyl esters) are formed during the must fermentation in order to preserve the organolectic quality of the wine [35,36,38].

## 3. Conclusions

The UV-denaturation assay has been developed at the high photon flux Diamond B23 beamline for SRCD to assess the relative protein stability and determine qualitatively ligand-binding interactions, in particular, for ligands devoid of UV chromophores or with little UV absorption. 

The UV-denaturation assay has been used successfully for the identification of stabilising agents in protein formulations, particularly for monoclonal antibodies. In general, protein–ligand binding interactions can be assessed monitoring the spectral changes of the CD of the protein upon ligand addition. The novel method of identifying ligand binding is to determine the change of the relative UV denaturation rate of the protein with and without ligand using B23 beamline. This assay is often coupled with the temperature study, another method used to determine molecular interactions that can be inconclusive due to the thermal effect associated with the displacement of the bound solvent molecules being cancelled, thus the ligand interaction is essential in revealing otherwise elusive protein–ligand binding interactions.

In summary, the protein UV-denaturation assay using the high photon flux Diamond B23 beamline for SRCD is an important tool that can be used to characterise the photostability of proteins as a function of the environment such as formulations, pH, concentration, excipients, chemical agents, detergents, membranes, and ligands/drugs.
